# Ectopic expression of lncRNA MVIH as a potential diagnostic biomarker in cervical cancer

**DOI:** 10.18632/genesandcancer.224

**Published:** 2022-11-23

**Authors:** Mohammad Ghanbari, Aida Aghazadeh, Elaheh Malekabbaslou, Ali Rajabi, Aref Sobhkhizy, Melika Maydanchi, Ali Saber, Reza Safaralizadeh

**Affiliations:** ^1^Department of Animal Biology, Faculty of Natural Sciences, University of Tabriz, Tabriz, Iran; ^2^Zimagene Medical Genetics Laboratory, Hamedan, Iran

**Keywords:** MVIH, lncRNA, non-coding RNA, biomarker, cervical cancer

## Abstract

Aim: Cervical cancer (CC) is one of the most common cancers in women. Recent advances in screening and vaccination against the papilloma virus (HPV) have increased protection against CC. However, there is no effective diagnostic biomarker and treatment approach during the course of the disease. The current study is thus aimed to evaluate the changes in the expression of lncRNA associated with microvascular invasion in hepatocellular carcinoma (lncRNA MVIH) and its diagnostic value as a biomarker in CC patients.

Materials and Methods: One-hundred and fifteen (*n* = 115) pairs of CC primary tumor and marginal non-tumor tissue samples were obtained from Tabriz Valiasr International Hospital (Tabriz, Iran). RNA extraction and cDNA synthesis followed by quantitative reverse transcriptase PCR (qRT-PCR) were considered to investigate alterations in the expression levels of MVIH in patients with CC. The associations between MVIH expression changes and clinicopathological features as well as its potential as a diagnostic biomarker were assessed using SPSS and GraphPad prism software and the receiver operating characteristic (ROC).

Results: The expression levels of MVIH were significantly higher in CC tumors as compared to marginal non-tumor samples (*p* < 0.0001). Overexpression of MVIH was significantly associated with younger age (*p* = 0.033), lymph node metastasis (*p* = 0.031), tumor invasion depth (*p* = 0.035), and squamous cell type of CC (*p* = 0.019). The ROC analysis for MVIH as a diagnostic biomarker revealed the respective sensitivity and specificity of 67.83 and 80.

Conclusions: Overexpression of MVIH in CC tumors suggests its oncogenic role during tumorigenesis. Thus, it may serve as a potential diagnostic biomarker.

## INTRODUCTION

CC is the second most common cancer and the fourth malignant cause of death in women worldwide [1]. Infection with HPV is the most important risk factor for CC. Other factors such as multiple sexual partners, marriage before the age of 18, smoking, and the use of oral contraceptive pills can raise the risk of CC. However, genetic susceptibility has been estimated to be lower than 1% [1, 2].

The origin of CC is the epithelium of the uterine cervix, particularly the squamocolumnar junction of the ectocervix and endocervix. Histologically, CC is divided into two main groups including squamous cell carcinoma (95%) and adenocarcinoma (5%) [3]. CC can be largely preventable by regular screening methods such as histologically checking epithelial cells and HPV testing on samples obtained by pap smears [4]. In addition, vaccination against HPV can significantly decrease the incidence of CC [5]. Radiotherapy and chemotherapy are the main treatments in patients with CC. However, these treatments are not always effective as they are associated with severe side effects and relapse [6]. Also, due to lack of suitable diagnostic biomarkers during CC development, it has progressed to invasive stages in most of the patients, which has resulted in lower survival rate [7]. So far, most biomarkers that have been used in clinically diagnosis in CC, are proteins such as squamous cell carcinoma antigen (SCC), serum fragments of cytokeratin 19 (CYFRA 21-1), and cancer antigen-125 (CA 125). However, the specificity and sensitivity of these biomarkers in early detection of CC are low [8]. Therefore, the identification of novel molecular biomarkers and therapeutic targets could be of great importance to improve clinical outcomes in patients with CC. In recent years, lncRNAs have received more attention in cancer research because of their tissue specificity as well as their role in tumorigenesis [9].

lncRNAs are more than 200 nucleotides long and do not encode proteins. They regulate the expression of critical genes through several biological processes at epigenetic, transcription, and post transcription levels [9]. The expression pattern of lncRNAs are more specific in cells and tissues than mRNAs and also, lncRNAs are easily found in body fluids due to their stability [10, 11]. Furthermore, lncRNAs have critical roles in regulating genes involved in cell cycle, apoptosis, angiogenesis, and metastasis [12]. All of these features make lncRNAs suitable biomarkers of diagnostic and prognostic biomarkers in many cancers so that dysregulation of lncRNAs in tumor tissues is associated with tumor type and stage. For example, lncRNAs such as HOXA-AS2, FOXD2-AS, and KRT18P55 are upregulated in gastric cancer (GC) and they are significantly associated with clinical parameters such as tumor size, lymph node metastasis and *H. pillory* infection, introducing them potential tumor markers for GC [13–15]. In addition, lncRNAs such as HOTAIR, H19, and MALAT1 have oncogenic roles since their upregulation in CC tumor cells leads to excessive cell proliferation and migration [16–18]. Moreover, other lncRNAs such as lncRNA MEG3 and lncRNA GAS5 have tumor-suppressing roles and they are downregulated during the development of CC [19, 20]. Analysis of diagnostic and prognostic value of lncRNA MEG3 showed it can be a potential biomarker in CC [21]. Also, downregulation of GAS5 predicts poor prognosis of patients with CC [22]. These studies show lncRNAs by having oncogenic and tumor suppressor roles, may be used as diagnostic and prognostic biomarkers to improve clinical outcomes in patients with CC, therefore detection of new lncRNAs as biomarkers of CC is valuable in diagnosis of CC in early stages [9].

lncRNA MVIH was first identified in hepatocellular carcinoma (HC). Its overexpression is associated with a poor prognosis, increased angiogenesis, and HC aggressiveness [23]. Moreover, dysregulation of MVIH can serve as a prognostic biomarker in non-small cell lung cancer (NSCLC) and breast cancer [24, 25]. However, the expression pattern of MVIH in CC has not been fully elucidated. This research is aimed to evaluate the expression levels of MVIH in CC tumors to assess the potential of this lncRNA as a diagnostic biomarker in CC patients.

## RESULTS

### Patient characteristics

One-hundred and fifteen (115) consecutive patients with CC were included in this study. The mean age of the patients was 50.93 (SD ± 10.20 years). Fifty-five (49.6%) patients were younger than 50 years old while the remaining (50.4%) were older than 50. Eighty-three (72.2%) patients had squamous cell carcinoma and 32 (27.8%) had adenocarcinoma subtype. Approximately, 31.3% of patients had poorly differentiated tumors, followed by 58.3% with moderate and 10.4% with well-differentiated tumors. The invasion depth of the tumor in the cervix was more than 2.3 cm in 28 (24.4%) patients and the remaining (75.6%) had a depth lower than 2.3 cm. In 57 (49.6%) patients, the size of the tumor was more than 5 cm. Approximately, 25.2% (29) of patients had lymph node metastasis. According to the TNM staging, 87 (75.6%) patients were in stage I/II, whereas 28 (24.4%) cases were in stage III/IV (Table 1).

**Table 1:** Association between MVIH expression and clinicopathological features in patients with cervical tumor

Variable	Number of patients [%]	MVIH mean expression (±SD) in tumor samples	MVIH mean expression (±SD) in non-tumor samples	*P*-value
**Age (years)**				0.033
≥50	58 [50.4]	0.0046 (0.002)	0.0031 (0.002)	
<50	57 [49.6]	0.0053 (0.002)	0.0030 (0.001)	
**Histology**				0.019
Squamous cell carcinoma	83 [72.2]	0.0052 (0.002)	0.0030 (0.001)	
Adenocarcinoma	32 [27.8]	0.0043 (0.002)	0.0032 (0.002)	
**Differentiation**				0.851
Poor	36 [31.3]	0.0051 (0.002)	0.0029 (0.002)	
Moderate	67 [58.3]	0.0049 (0.002)	0.0032 (0.001)	
Well	12 [10.4]	0.0048 (0.002)	0.0026 (0.002)	
**Tumor size (cm)**				0.840
≥5 cm	57 [49.6]	0.0049 (0.002)	0.0030 (0.001)	
<5 cm	58 [50.4]	0.0050 (0.002)	0.0031 (0.002)	
**TNM stage**				0.188
I/II	87 [75.6]	0.0051 (0.002)	0.0031 (0.001)	
III/IV	28 [24.4]	0.0046 (0.001)	0.0029 (0.001)	
**Lymph node Metastasis**				0.031
Absent	86 [74.8]	0.0047 (0.002)	0.0031 (0.002)	
Present	29 [25.2]	0.0056 (0.002)	0.0028 (0.001)	
**Tumor invasion depth (cm)**				0.035
≥2.3	28 [24.3]	0.0056 (0.002)	0.0028 (0.001)	
<2.3	87 [75.7]	0.0047 (0.002)	0.0031 (0.001)	

### The expression of MVIH in CC tumor

**Figure 1:**
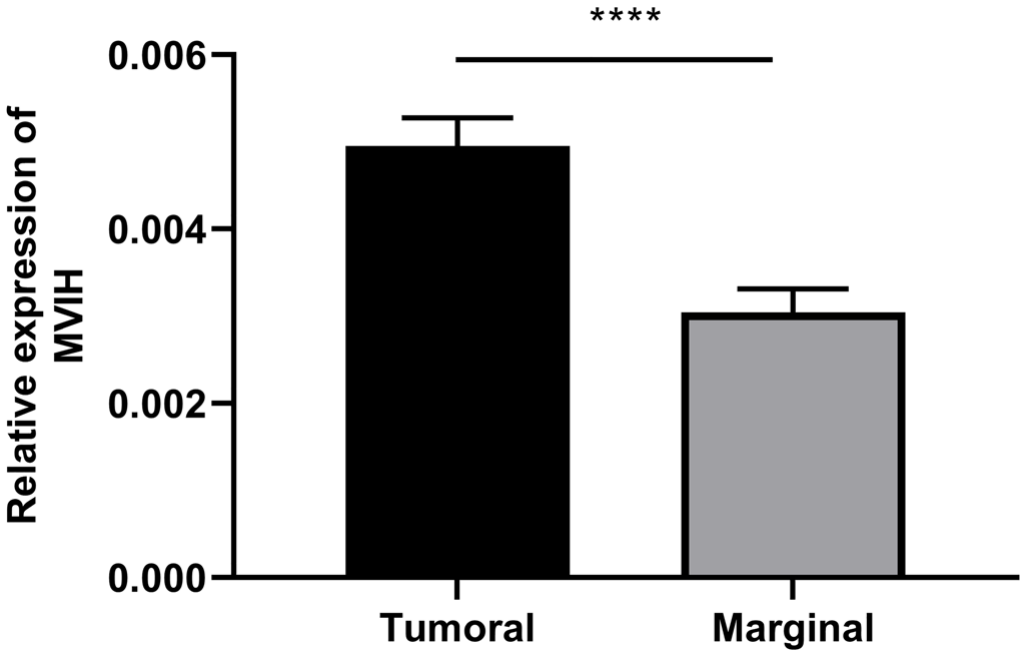
Expression of lncRNA MVIH in CC tumor as compared to marginal non-tumor tissues. ^****^p-value < 0.0001.

qRT-PCR was performed to assess the expression of MVIH in CC primary tumors relative to marginal non-tumor tissue. Our results revealed that the expression of MVIH was significantly higher in the tumor as compared to non-tumor tissues (*p* < 0.0001) (Figure 1).

### Association between MVIH expression levels and clinicopathological features

The overexpression of MVIH was significantly associated with the patients’ age (younger than 50 years old) (*p* = 0.033). A significant positive relationship was detected between upregulation of MVIH and lymph node involvement (*p* = 0.031). The expression of MVIH was significantly higher in tumors with deeper invasion into the cervix (*p* = 0.035). Furthermore, MVIH was significantly overexpressed in the squamous cell subtype compared to the adenocarcinoma subtype (*p* = 0.019). No significant association was observed between elevated levels of MVIH and other clinicopathological features such as tumor size, tumor differentiation, and disease stage (Table 1).

### MVIH expression as a potential biomarker of CC

**Figure 2:**
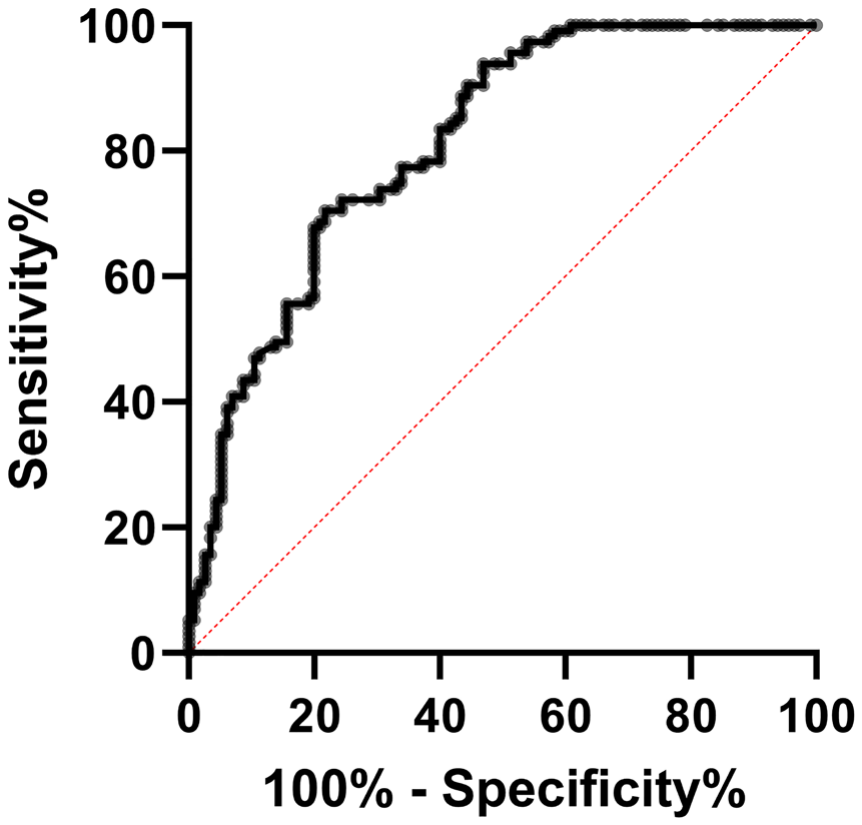
The ROC curve analysis showed a sensitivity and specificity of 67.83 and 80, respectively. AUC = 0.8114.

**Figure 3:**
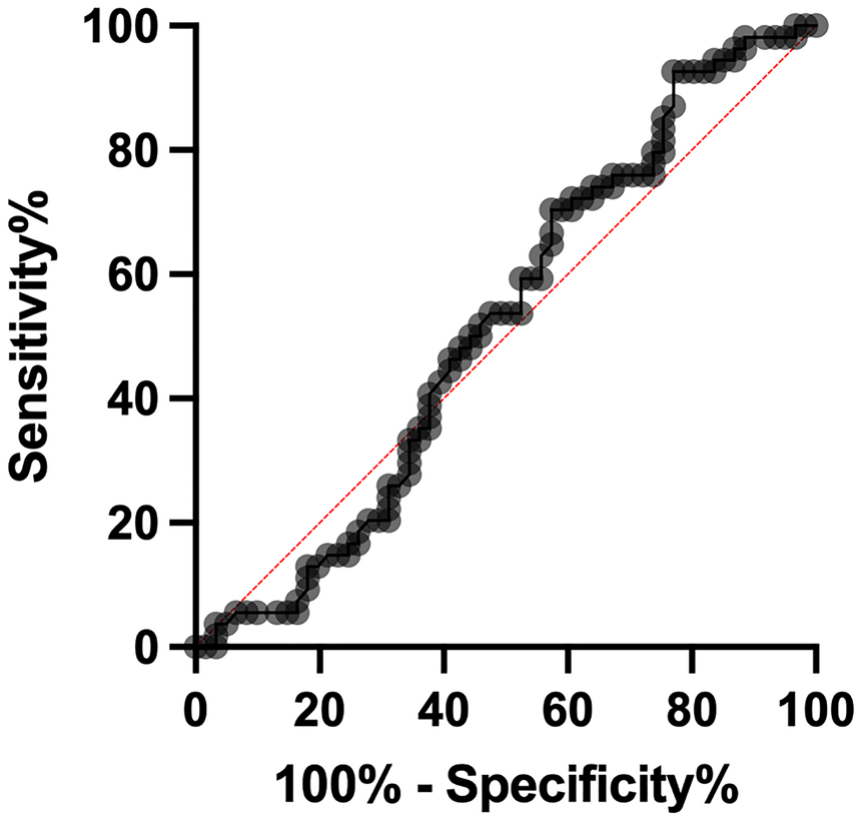
The ROC curve analysis of distant metastasis showed sensitivity and specificity of 53.70 and 52.46 respectively. AUC = 0.5213.

The ROC analysis was employed to examine MVIH potentials as a diagnostic biomarker for CC. Our analysis revealed a sensitivity and specificity of 67.83 and 80, respectively. The area under curve (AUC) was 0.8114 and the cut-off value was set to 0.0039 (Figure 2 and Table 2). Also, the ROC analysis of distant metastasis revealed MVIH as a weak prognostic biomarker with sensitivity and specificity of 53.70 and 52.46 respectively. The AUC was 0.5213 and the cut-off value was <0.004804 (Figure 3 and Table 3).

**Table 2:** The statistical analysis of the ROC curve for MVIH in cervical cancer

The ROC curve data	Values
The area under the ROC curve	0.8114
Sensitivity (%)	67.83
Specificity (%)	80
Cutoff score	>0.0039
Std. error	0.02784
95% confidence interval	0.7569–0.8660
*P*-value	<0.0001
Number of tumor tissue specimens	115
Number of non-tumor tissue specimens	115

**Table 3:** The distant metastatic statistical analysis of the ROC curve for MVIH in cervical cancer

The ROC curve data	Values
The area under the ROC curve	0.5213
Sensitivity (%)	53.70
Specificity (%)	52.46
Cutoff score	<0.004804
Std. error	0.05422
95% confidence interval	0.4150–0.6275
*P*-value	0.6949
Number of metastatic patients	61
Number of non-metastatic patients	54

## DISCUSSION

In the present study, lncRNA MVIH expression levels were assessed in CC tumors as compared to marginal non-tumor samples. MVIH was significantly upregulated in CC tumor tissue. Furthermore, overexpression of MVIH was significantly associated with younger age, squamous cell subtype, lymph node metastasis, and tumor depth of invasion.

The overexpression of MVIH was found in CC tumors in comparison with non-tumor tissues which is consistent with previous studies on other types of cancer, supporting the oncogenic role of this lncRNA in cancer. MVIH imposes an oncogenic mechanism by stimulation of several pathways with crucial roles in angiogenesis, cell proliferation, invasion, and apoptosis resistance [23]. However, the answer to the question of how MVIH plays such an oncogenic role in CC tumorigenesis requires deeper functional studies.

Our finding revealed a significant association between MVIH overexpression and depth of invasion and lymph node metastasis which is consistent with findings in NSCLC and glioblastoma; where MVIH overexpression is correlated with metastasis and tumor invasiveness. MVIH upregulation promotes invasion of NSCLC cells via matrix metalloproteinase 2 and 9 (MMP2/MMP9) expression *in vitro* [24]. In glioma, the overexpression of MVIH was associated with cancer cell proliferation, invasion, and migration [26]. In addition, MVIH is involved in the upregulation of *AKT* and *CXCR4* by sponging miR-302a leading to cell proliferation and invasion in glioblastoma [27]. MVIH also plays a decisive role in cell proliferation, angiogenic, and anti-apoptotic pathways. Yuan et al. (2012) showed that MVIH expression leads to angiogenesis in HC via inhibition of an anti-angiogenic protein known as PGK1 [23]. On the other hand, Shi et al. (2015) revealed that MVIH inhibits apoptosis in HC cells and stimulates cell growth through modulating miR-199a expression [28].

Overexpression of MVIH in breast cancer and acute myeloid leukemia (AML) cells resulted in increased cell proliferation and inhibited apoptosis, while MVIH knockdown suppressed cell proliferation and enhanced apoptosis [25, 29]. In addition, the expression of MVIH is correlated with Ki67 expression in breast cancer. The expression of nuclear antigen Ki67 is a proliferative index of cycling cells [25, 30, 31]. Another study revealed that MVIH overexpression led to increased cell proliferation and resistance to apoptosis in AML cells via sponging miR-505. In this mechanism, by blocking miR-505, MVIH triggers the upregulation of oncogenic genes including *HMGB1* and *CCNE2* which have important roles in cell proliferation and resistance to apoptosis [32]. Despite extensive studies on different cancer types, angiogenic and anti-apoptotic roles of MVIH in CC have not been clarified and more precise functional studies are required. However, the mediatory role of MVIH in cancer cell invasiveness makes it a potential therapeutic target in cancer therapy.

lncRNAs are strictly regulated in cells and show cell type and tissue specifications. They play important roles in diverse biological processes and their dysregulation has been reported in a wide range of malignancies, suggesting them as high-potential biomarkers [33]. lncRNAs could be suitable biomarkers because they are stable, tissue-specific, and easily detectable in body fluids [34]. Several studies have shown the prognostic potential of MVIH as a marker in different cancer types such as AML, gastric, glioma, and breast cancers [26, 29, 35, 36]. Similarly, this study indicated that MVIH can be used as a potential diagnostic biomarker in CC patients. However, it seems that MVIH could be a weak prognostic biomarker in metastasis to lymph node.

## MATERIALS AND METHODS

### Tissue specimens

One hundred and fifteen pairs of CC tissue and marginal non-tumor tissue samples were collected from Tabriz Valiasr International Hospital (Tabriz, Iran). The Medical Ethics Committee of the University of Tabriz approved the study (approval number: IR. TABRIZU. REC. 1398.015). Written informed consent was obtained from patients. This study was conducted in compliance with the provisions of the Declaration of Helsinki and Good Clinical Practice guidelines. Tissue specimens were collected and immediately placed in liquid nitrogen. All frozen samples were stored at −80°C. An experienced pathologist examined and determined the histopathological characteristics of the specimens.

### RNA isolation and cDNA synthesis

Total RNA was isolated from samples using TRIZOL reagent based on the manufacturer’s protocol (Invitrogen, Waltham, MA, USA). RNA samples were treated with DNaseI (GeneAll, Seoul, Korea) to remove DNA contamination. RNA was quantitatively and qualitatively examined using a nanodrop (Thermo Fisher Scientific, Waltham, MA, USA) and on 3% (w/v) agarose gel electrophoresis, respectively.

Approximately, 500 ng total RNA was used as the template for cDNA synthesis using the Takara cDNA synthesis kit (Takara, Kusatsu, Japan) according to the manufacturer’s instructions. All RNA and cDNA samples were stored at −80°C.

### qRT-PCR

PCR primers are listed in Table 4. qRT-PCR was performed using SYBR Green Master Mix (Amplicon, Odense, Denmark) and a Light Cycler® 96 Real-Time PCR system (Roche Molecular Systems, Pleasanton, CA, USA). The total volume of each reaction was 14 μl comprising 7 μl of SYBR Green Master Mix (2×), 0.6 μl of specific primers for MVIH (10 μM) and β-actin (10 μM), 1 μl of cDNA (100 ng/μl), and 5.4 μl ddH_2_O. The thermal cycling program was as follows: step 1: 95°C for 10 min, step 2: 40 cycles including 95°C for 30 sec and 60°C for 30 sec, and 72°C for 30 sec, and step 3: 72°C for 5 min. Each experiment was carried out in triplicate and relative MVIH expression was evaluated using the comparative cycle threshold (Ct) method. MVIH gene expression levels were then normalized to β-actin expression levels and the difference between MVIH and β-actin Ct values (ΔCt) was calculated for each sample. Finally, MVIH expression levels were determined in tumor versus non-tumor tissues by calculating 2^−ΔCt^.

**Table 4:** Designed RT-PCR primers

Gene	Forward primer sequence	Reverse primer sequence
lncRNA MVIH	5′-AATTTTGCACATCTGAACAGCC-3′	5′-TTCAAAATCCCACTACGCCCA-3′
β-actin	5′-AGAGCTACGAGCTGCCTGAC-3′	5′-AGCACTGTGTTGGCGTACAG-3′

### Statistical analysis

SPSS version 26 software (SPSS Inc., Chicago, IL, USA) and GraphPad Prism Version 9.0 (GraphPad software, San Diego, CA, USA) were used for statistical analysis. Mann-Whitney and one-way ANOVA tests were used to analyze the association between MVIH expression levels and clinicopathological features. The ROC analysis was also conducted to determine the sensitivity and specificity of MVIH as a biomarker in patients with CC. The confidence interval (CI) was 95% and *p*-values smaller than 0.05 were considered statistically significant.

## CONCLUSIONS

In conclusion, MVIH is significantly overexpressed in CC tumors in comparison with non-tumor tissues. The overexpression of MVIH is associated with tumor invasion depth and lymph node metastasis, it can be also considered a potential diagnostic biomarker in patients with CC to improve their clinical outcomes.
